# Tissue Engineering and Biomaterial Strategies to Elicit Endogenous Neuronal Replacement in the Brain

**DOI:** 10.3389/fneur.2020.00344

**Published:** 2020-04-28

**Authors:** Erin M. Purvis, John C. O'Donnell, H. Isaac Chen, D. Kacy Cullen

**Affiliations:** ^1^Center for Brain Injury & Repair, Department of Neurosurgery, Perelman School of Medicine, University of Pennsylvania, Philadelphia, PA, United States; ^2^Center for Neurotrauma, Neurodegeneration & Restoration, Corporal Michael J. Crescenz Veterans Affairs Medical Center, Philadelphia, PA, United States; ^3^Department of Bioengineering, School of Engineering and Applied Science, University of Pennsylvania, Philadelphia, PA, United States

**Keywords:** neural precursor cells, neuroblasts, adult neurogenesis, subventricular zone, tissue engineering, biomaterials, neural regeneration

## Abstract

Neurogenesis in the postnatal mammalian brain is known to occur in the dentate gyrus of the hippocampus and the subventricular zone. These neurogenic niches serve as endogenous sources of neural precursor cells that could potentially replace neurons that have been lost or damaged throughout the brain. As an example, manipulation of the subventricular zone to augment neurogenesis has become a popular strategy for attempting to replace neurons that have been lost due to acute brain injury or neurodegenerative disease. In this review article, we describe current experimental strategies to enhance the regenerative potential of endogenous neural precursor cell sources by enhancing cell proliferation in neurogenic regions and/or redirecting migration, including pharmacological, biomaterial, and tissue engineering strategies. In particular, we discuss a novel replacement strategy based on exogenously biofabricated “living scaffolds” that could enhance and redirect endogenous neuroblast migration from the subventricular zone to specified regions throughout the brain. This approach utilizes the first implantable, biomimetic tissue-engineered rostral migratory stream, thereby leveraging the brain's natural mechanism for sustained neuronal replacement by replicating the structure and function of the native rostral migratory stream. Across all these strategies, we discuss several challenges that need to be overcome to successfully harness endogenous neural precursor cells to promote nervous system repair and functional restoration. With further development, the diverse and innovative tissue engineering and biomaterial strategies explored in this review have the potential to facilitate functional neuronal replacement to mitigate neurological and psychiatric symptoms caused by injury, developmental disorders, or neurodegenerative disease.

## Introduction

Neurogenesis in the mammalian brain is a continuous process that occurs in multiple stages throughout the developing brain (1, 2) and persists into adulthood (3, 4). Interest in adult neurogenesis in the human brain has gained significant momentum over the past several years. Research in the field of neurogenesis has included basic investigations of the neurogenic potential of the brain over time as well as how this capacity can be harnessed as a neuronal replacement strategy throughout one's lifetime following brain injury and/or neurodegenerative disease. Previous observations that endogenous neurogenesis can become altered in disease states (5–8) have led to investigations of how biomaterials and tissue engineering can be utilized to augment neurogenesis in the adult brain (9–13). While these emerging strategies may have the potential to promote neuronal replacement, there are still several challenges to overcome before these technologies can be successfully utilized in application to injury and disease.

There is controversial evidence regarding whether adult neurogenesis continues in the human subventricular zone (SVZ) surrounding the lateral ventricles and in the subgranular zone (SGZ) of the hippocampal dentate gyrus (DG) throughout life (3, 14–17). Of particular interest in this review is the possibility that neural precursor cells (NPCs) exist in some quantity in the SVZ throughout the human lifespan. While there is considerable debate regarding the quantity of NPCs that exists within this neurogenic niche and the existence and functionality of the rostral migratory stream (RMS) as the human brain matures and ages (reviewed in section Controversy Over Human SVZ and RMS below), there is evidence from several different groups indicating that the adult human SVZ contains some quantity of NPCs (18–22).

The current evidence indicating regionally-restricted locations of NPCs within the adult brain (3, 23) and the propensity of adult SVZ NPCs to mature into an interneuron phenotype (24) limits their potential to contribute to self-repair. The various brain injuries and neurodegenerative diseases that can cause the loss of diverse neuronal types across distinct brain regions urgently calls for the development of reliable methods for diverse neuronal replacement. The advent of advanced regenerative medicine therapies has led to the production of various biomaterial-, cell-, and/or microtissue-based methods that could be utilized in an attempt to promote or enhance neuronal replacement across various brain regions.

There are currently three general strategies to replace lost neuronal populations throughout the brain: exogenous stem cell transplants (25–29), direct cell reprogramming (30–35), and redirection of endogenous NPCs (5, 36). Here, we specifically review pharmacological, biomaterial, and tissue engineering strategies that have been developed in an attempt to harness the neurogenic potential of the SVZ to serve as an endogenous, reliable NPC source to replace lost or damaged neurons throughout life. While some strategies rely on the application of extrinsic factors to modulate neurogenesis within the SVZ, others manipulated the intrinsic properties of the SVZ to redirect endogenous NPCs. Pharmacological strategies include the use of neurotrophic factors and signaling peptides to augment NPC proliferation. More recent research has also led to the fabrication of various types of acellular scaffolds and hydrogels designed to facilitate or divert immature neuroblast migration from the SVZ (37). In addition, we review an exogenously biofabricated tissue-engineered RMS developed by our laboratory that replicates the structure and function of the endogenous glial tube (38–40). This novel replacement strategy seeks to redirect endogenous neuroblast migration from the SVZ through an engineered “living scaffold” and to diverse, specific locations throughout the brain. This microtissue construct, unique from acellular biomaterial scaffolds in that it is comprised of living cells, is the first tissue-engineered neuronal replacement strategy that attempts to replicate and expand upon one of the brain's intrinsic mechanisms for neuronal replacement.

The strategies discussed in this review have the potential to enhance neuronal replacement in response to a wide range of brain disorders, including acquired brain trauma, developmental disorders, and neurodegenerative diseases. As such, the current article presents the technical details and findings to date for a range of regenerative therapies designed to facilitate endogenous neuronal replacement. Importantly, we discuss several challenges that need to be overcome in order to better utilize endogenous stem cells for repair purposes and discuss the diseases and disorders across the human lifespan for which these emerging technologies may be particularly applicable. Before we can reliably utilize these biomaterials and tissue engineering strategies as safe, effective clinical therapies to enhance neuronal regeneration, continued advancements must be made to ensure neuronal survival, reliable maturation and differentiation into appropriate cell types in target regions, proper integration, and network formation with resident neurons, and development of reliable ways to measure and modulate these factors. In particular, we recognize that the ability to influence cell fate determination is currently a major limitation of all of the strategies discussed herein. We attempt to answer what we can regarding the potential of these strategies with the understanding that success will be determined by future research indicating their ability to influence cell determination. Moreover, we consider the particular challenges in employing these new technologies as treatments to diseases and disorders that are not fully understood mechanistically. These challenges are critical to contemplate as we move toward utilization of these technologies in clinical trials. Overall, biomaterial and tissue engineering strategies that can reliably direct and enhance neurogenesis in a sustained fashion and to diverse brain regions may have the potential to successfully promote neuronal replacement in quantities large enough to effectively enhance functional recovery following a variety of injury, degeneration, and disease states that arise throughout human life.

## Neurogenesis in the Adult Brain

Neurogenic niches remain in some form within the mammalian brain post-development (3, 14, 41, 42). Here, we review the location of these niches and discuss the unresolved controversy over the existence and function of the SVZ and RMS in the adult human brain. We also review observations of altered NPC production and neuroblast migration in the mammalian brain following diverse types of brain injury.

### Endogenous Stem Cell Sources in the Postnatal Brain

There is ongoing discussion in the field of neuroscience regarding the exact definitions of neural stem and progenitor cells. Differentiation of stem cells exists along a continuum and multiple terms are currently used to define these different types of immature cells. In this review, we use the term neural precursor cells (NPCs), which is a broad term that encompasses both neural stem and progenitor cells. We recognize that by using this umbrella term we may lose some of the nuances of the particular cell types discussed in the literature reviewed herein. However, we choose to use this term for the sake of clarity and conciseness. With regards to the SVZ, we use the term NPCs when referring to immature cells from the time that they are activated type B1 cells in the SVZ to the time that they become type A cells (neuroblasts). We use the term neuroblast to refer to immature neurons that are migrating. We recognize that there is further detail and nuance surrounding these terms that will not be discussed in this review.

The idea that neurogenesis in mammals is restricted to embryonic development was first challenged by Joseph Altman in the 1960's when he demonstrated radioactive thymidine incorporation in cerebral neurons of adult rats (43, 44). Subsequent electron microscopy analysis provided evidence that that these newborn cells in the brain are indeed neurons (45) and demonstrated that they are capable of integrating into functional neuronal circuits (46). *In vitro* research then demonstrated that cells isolated from the adult mouse brain are capable of dividing and differentiating into astrocytes and neurons (47, 48), suggesting the existence of NPCs in the postnatal brain. As exploration of adult neurogenesis progressed, research began to focus on factors that control this NPC proliferation and neuroblast migration, including neurotransmitters (49, 50), receptors (51), signaling RNAs (52), neurotrophic factors (53, 54), and cell intrinsic signaling molecules (55, 56).

**Figure 1:**
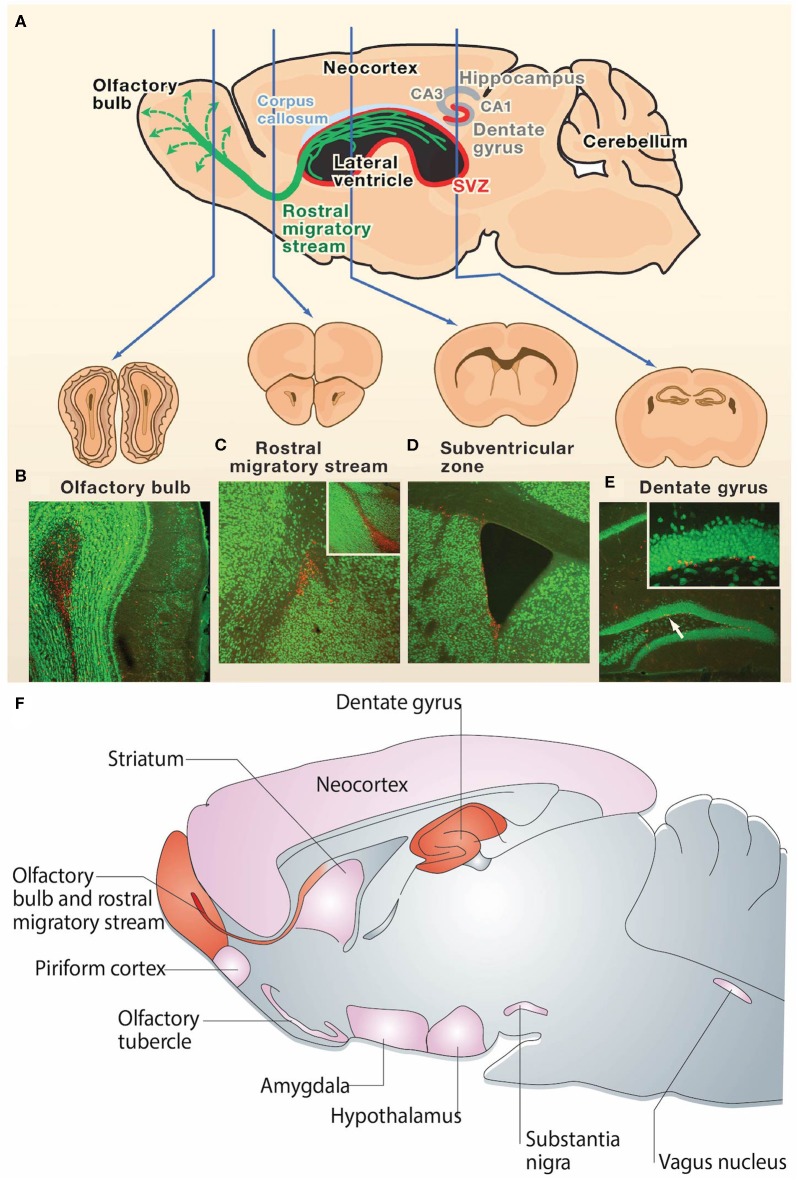
Neurogenesis in the adult rodent brain. **(A)** Neurogenesis occurs post-development in the rodent brain in the subgranular zone of the hippocampal dentate gyrus and the subventricular zone surrounding the lateral ventricles (neurogenic regions depicted in red). Immature neurons born in the subventricular zone migrate along the rostral migratory stream (green) to the olfactory bulb. **(B–E)** NeuN (green) and BrdU (red) staining in coronal sections of the **(B)** adult mouse olfactory bulb, **(C)** rostral migratory stream, **(D)** subventricular zone, and **(E)** dentate gyrus. The presence of BrdU is indicative of post-developmental neurogenesis in these 4 regions. The inset in **(C)** is a sagittal view of the rostral migratory stream and the inset in **(E)** is the dentate gyrus at higher magnification. **(F)** Sagittal view of the rodent brain depicting neurogenic regions. Regions in which adult neurogenesis has been repeatedly shown to occur are depicted in red, and regions in which there is controversial evidence of low levels of adult neurogenesis are depicted in pink. Reprinted with permission from Zhao et al. (23) for **(A–E)** and Gould et al. (62) for **(F)**.

The discovery of NPCs in the SGZ of the DG and the SVZ surrounding the lateral ventricles has emerged as a reproducible finding in the brains of most mammals (3, 23, 57–59). New NPCs generated in the SGZ mature into excitatory cells in the DG and form synapses with target cells in the hilus and area CA3 (60, 61) (Figures 1A,E). Simultaneously, neuroblasts formed in the SVZ migrate through the RMS to the olfactory bulb (OB), where they mature into inhibitory granule and periglomerular neurons and form functional synaptic connections (24) (Figures 1A–D). These newly formed neurons generated from both neurogenic niches serve a wide variety of functional purposes in the mammalian brain, including cognitive functions such as odor memory, perceptual learning, and mate recognition (63–65). Additionally, there is evidence showing the presence of neurogenesis in other brain regions such as the neocortex, corpus callosum, striatum, amygdala, septum, piriform cortex, olfactory tubercle, thalamus, hypothalamus, and substantia nigra of adult mammals, including rats, mice, hamsters, rabbits, and macaques (62, 66, 67) (Figure 1F). However, adult neurogenesis within the human brain remains controversial. Divergent evidence recently emerged regarding the existence of adult neurogenesis in the human hippocampus (15–17). While some studies suggest that hippocampal neurogenesis continues throughout human life (68–70), competing evidence argues that hippocampal neurogenesis ceases during childhood (71, 72). There is currently insufficient evidence to conclude whether hippocampal neurogenesis continues throughout adulthood in humans (15). Additionally, there is lack of consensus regarding various aspects of adult neurogenesis within the human SVZ (3, 14), which will be discussed in detail in section Controversy Over Human SVZ and RMS.

Of particular interest to this review is neurogenesis arising from the SVZ. There has now been consensus for several decades that NPCs continue to exist in the SVZ throughout the life of fish, reptiles, and several different types of mammals (73–79). The rodent SVZ has been the most extensively studied and is the most well-characterized. The neurobiology of the rodent SVZ and the factors regulating SVZ neurogenesis have been recently reviewed by the Alvarez-Buylla group and will not be discussed here (78, 80). However, it is critical to consider the factors that control endogenous proliferation, differentiation, and migration of SVZ-derived NPCs, as well as how these factors change with aging (81), in order to understand whether endogenous SVZ neurogenesis may have the potential to be upregulated. Upregulation of SVZ-derived NPCs may be required in order to promote the success of the biomaterial and tissue-engineered technologies discussed in section Current Strategies to Augment and Redirect Endogenous Neurogenesis. Here, there is substantial evidence regarding the cell cycle and lineage tracing of SVZ-derived NPCs, as well as factors that play an important role in regulating SVZ-derived NPC proliferation in the adult rodent brain (78, 80, 82–84). Research suggests that the majority of rodent hippocampal NPCs divide asymmetrically and that there may be a limited number of proliferative cycles emerging from the rodent SGZ (85–88). In contrast, recent research has demonstrated that the majority of SVZ-derived NPCs divide symmetrically and that ~20% of these B1 cells symmetrically self-renew, allowing neurogenesis to continue throughout adulthood (82). However, it remains unknown whether there is a limited number of times that B1 cells can symmetrically self-renew. This concept should be more thoroughly investigated as it will shed light on whether there is a limited number of proliferative cycles of SVZ-derived NPCs, a factor that will be important to consider for the success of the biomaterial and tissue-engineered technologies reviewed in section Current Strategies to Augment and Redirect Endogenous Neurogenesis.

The structure of the SVZ and mechanisms of adult neurogenesis arising from this region are highly heterogeneous among species, even between mammalian species (89, 90). In addition to differences in structure and organization of the SVZ and RMS, NPC maturation, survival, migration, and circuit integration of cells arising from the SVZ also differ among species (89). While certain mammalian species such as rodents seem to have detectable and continual SVZ neurogenesis throughout life, production of neuroblasts within the adult human SVZ may be more sparse (19, 67). Due to the challenges associated with performing neurogenesis research in the human brain, the majority of previous research on adult neurogenesis has been conducted in model organisms such as rodents and birds. However, it is essential to keep in mind that evolutionary and ecological adaptations among species contribute to differences in the mechanisms of adult neurogenesis (90, 91). Evidence elucidating mechanisms of adult neurogenesis that is gathered from basic research conducted in model organisms cannot necessarily be extrapolated to humans. Further post-mortem and neuroimaging research within the brains of humans and other mammalian species with gyrencephalic brains is therefore needed before conclusions regarding the exact neurogenic mechanisms regulating the adult SVZ in higher-order mammals can be reached.

### Controversy Over Human SVZ and RMS

Divergent evidence exists regarding the existence and structure of the SVZ and RMS as the human brain matures. While it appears that the human SVZ continues to exist within the adult brain and retains some level of neurogenic potential (18–22), there are competing findings concerning how the quantity of NPCs produced within the SVZ changes over time. Here we will discuss the unresolved debate over the existence and structure of the RMS, whether NPCs exist within the RMS, and where these NPCs end up if they are indeed being produced and migrating within the adult human brain.

Evidence gathered from analysis of ten necropsied OBs from healthy postmortem human patients supported the notion that NPCs in the human brain migrate from the SVZ to the OB and continuously populate the OB throughout life (92). In the same year, Alvarez-Buylla and colleagues described the structure of the human SVZ as containing ribbon of proliferative astrocytes that surround the wall of the lateral ventricles and are separated from the ependyma by a process-filled gap (18). After analyzing human SVZ specimens from over 100 neurosurgical resections and autopsied brains, they found evidence that these SVZ astrocytes are capable of neurogenesis, but did not find evidence of migrating neuroblasts either within the adult SVZ or moving toward the OB (18). Further analysis by this group of just under 100 SVZ tissue specimens revealed small numbers of proliferating cells in the SVZ, providing further evidence that the adult human SVZ retains neurogenic potential, but again demonstrated no evidence of chains of migrating neuroblasts within the SVZ (93). The Eriksson lab analyzed sagittal forebrain sections gathered from three human patients and described the structure of the human SVZ as a ribbon of astrocytes surrounding the lateral ventricular walls. Additionally, and in contrast to previous research, they described the structure of the human RMS as extending along what they termed a lateral ventricular extension up to the olfactory bulb, and found evidence of large numbers of chains of migrating neuroblasts proliferating within this structure that go on to become mature neurons in the OB (20). In a comment on these findings, the Alvarez-Buylla lab contested that the high levels of proliferation observed in this study were not confirmed using different markers (i.e., Ki67, Tuj1, PSA-NCAM). They additionally disputed the presence of a ventricular extension and questioned the chains of migrating neurons that were claimed to have been found within it (94). The Eriksson lab stood by their findings, stating that their use of meticulous labeling with PCNA, BrdU, and NeuN and their rigorous MRI imaging combined with the use of serial sagittal sectioning made their study advantageous compared with previous studies that had been conducted by other groups. They argued that neurogenesis in the human OB is as robust as that of rodents, but that the human RMS is distinct in that it is oriented in a different plane than that of rodents, another factor that could have caused this structure to have gone unseen in previous examinations (95). A few years later, the Alvarez-Buylla lab analyzed specimens from 10 neurosurgical resections and 50 autopsied brains and found evidence of immature neuronal cells migrating in the human SVZ and RMS during early development, highlighting that they found an absence of evidence for such migration in older children and adults. They additionally demonstrated that neuroblasts from the SVZ migrate to both the prefrontal cortex and the OB during the first 18 months of life, after which SVZ neurogenesis drastically decreases (19). While this study did provide evidence for the presence of migrating neuroblasts in the juvenile RMS, it did not demonstrate a high volume of these migrating cells as was reported by Curtis and colleagues in 2007 (14, 19). Additional evidence gathered from the Yang lab's analysis of tissue samples from six adult human patients supported the findings that neuroblasts are present in the human SVZ and RMS throughout adulthood, but do not exist within the adult OB (21). Recently, single-cell RNA sequencing was used to identify pools of NPCs in adult human olfactory neuroepithelium gathered from patients undergoing endoscopic nasal surgery, providing further evidence for the continued presence of adult neurogenesis in the human brain (96).

Controversial evidence has also emerged regarding the migration of NPCs from the SVZ to regions of the brain other than the OB. The Frisen lab has gathered evidence demonstrating that neuroblasts produced in the SVZ mature into interneurons and integrate into the neighboring striatum, a process that continues to occur throughout adulthood (97). However, evidence gathered by the Yang lab contrasted these findings and showed that neuroblasts from the SVZ do not integrate into the striatum as mature interneurons (98). Other groups have suggested alternative sources other than the SVZ from which new neurons in the striatum may arise, including parenchymal astrocytes (14, 99).

As research collectively demonstrates, it remains unknown whether neurogenesis exists within the adult human SVZ, the extent to which this process occurs if it does exist, and the fate of potential NPCs arising from this region. Much of the divergent evidence presented above persists due to challenges associated with performing research on human tissue, as well as variation in the techniques and reagents utilized for tissue handling and staining. Many of these studies had small sample sizes and grouped together specimen samples gathered from heterogeneous human groups. Some of these studies analyzed brain tissue from healthy humans while others analyzed tissue from patients that possessed a wide variety of diseases and disorders prior to death. Patients who possessed non-neurological diseases and disorders may still have had altered or disrupted function of the SVZ. It is also important to consider how the levels of adult neurogenesis could intrinsically vary between different individuals, and how factors such as gender, exposure to various environmental factors, and lifestyle choices including diet and exercise could affect this phenomenon. The individual variability of subjects utilized in these studies makes it difficult to extrapolate staining from a handful of specimens to the entire human population.

### SVZ Responses to Acquired Brain Injury

There have been multiple reports of altered SVZ neurogenesis and modified migration patterns of SVZ-derived neuroblasts in response to various forms of acquired brain injury in rodents, non-human primates, and humans. These studies, discussed below, provide examples of attempted intrinsic repair mechanisms utilizing endogenous SVZ NPCs to replace lost or damaged neurons following acquired brain injury in adult mammals.

Experimental stroke induced by middle cerebral artery occlusion (MCAO) in adult rats caused increased proliferation of NPCs in the SVZ (100). These new neurons migrated to the damaged striatum where they matured into spiny neurons and formed synapses with existing striatal neurons (100). This process can continue for up to 4 months following MCAO (101). These NPCs appear to be generated in the SVZ rather than in the striatal parenchyma, and migrated in chains, guided by blood vessels, from the SVZ toward the injured striatum (102–104). Experimental stroke in rodents modulates the gene profiles and electrophysiological characteristics of NPCs within the SVZ, specifically by inducing hyperpolarized resting membrane potentials and increasing expression of tyrosine hydroxylase, both of which are markers of neuronal maturity (105). Additionally, neuroblasts generated post-stroke migrate to the injured cortex, although to a lesser extent than the migration toward the striatum (106, 107). External factors such as environmental enrichment further increase neurogenesis in the rodent SVZ post-stroke (108). Neuroblast migration through the RMS is facilitated by release of the soluble signaling molecule Slit1 from neuroblasts and detection by the Robo2 receptors of glial tube astrocytes (109). Neuroblasts overexpressing Slit1 migrated farther into a stroke-induced lesion compared to control neuroblasts in the mouse brain (110). These neuroblasts matured into striatal neurons, integrated into striatal circuitry, and improved functional recovery following MCAO (110).

Increased production and altered migration patterns of SVZ-derived neuroblasts have also been observed in rodent models of disease other than stroke. For example, augmented SVZ NPC production has been observed in a mouse model of Huntington's disease (HD) (111). These SVZ-derived neuroblasts migrated to the injured striatum, but did not demonstrate cell maturity or synaptic integration (111). Simultaneous lesioning of dopaminergic neurons in the substantia nigra and infusion of the transforming growth factor (TGF)-alpha into the striatum caused increased NPC production in the SVZ and migration of these cells toward the striatum (112). Immunoreactivity of cells for BrdU and tyrosine hydroxylase or BrdU and the dopamine transporter indicated that these NPCs reaching the striatum may have differentiated into dopamine neurons (112). Increased SVZ neurogenesis has also been reported in the rodent brain following experimental demyelination (113), targeted lesioning (114–117), lateral fluid percussion brain injury (118, 119), and controlled cortical impact brain injury (120–122). Additionally, global cerebral ischemia in adult macaques caused increased numbers of immature neurons in the SVZ (123).

Altered NPC production and neuroblast migration has also been observed following acquired brain injury in adult humans. Augmented neurogenesis has been reported post-stroke in regions surrounding infarct cortical tissue (124). Damaged tissue from postmortem stroke patients contains increased numbers of cells immunoreactive for Ki67, DCX, TuJ1, and TUC-4 compared to tissue from healthy controls (124). A later case study demonstrated increased neurogenesis following cerebrovascular infarction in the aging brain, showing the accumulation of NPCs, identified via positive staining for Nestin, Musashi, Sox2, PSA-NCAM, and SSEA-4, adjacent to the SVZ as well as in the area of brain injury (125). Increased NPC production has been observed in brain tissue of adult humans following subarachnoid hemorrhage caused by a ruptured aneurysm (126). Here, NPCs were identified by increased expression of nestin, vimentin, Sox2, Musashi1, Musashi2, and immunoreactivity for both Ki67 and Musashi2 in hemorrhage patients compared to controls (126). This study did not determine where these cells were generated and whether they matured into functional neurons (126). Increased proliferation of cells expressing PCNA, TuJ1, and GFAP in the SVZ has also been observed in adult patients with HD (127), and further research using carbon-14 dating approaches provided evidence that SVZ-derived neuroblast migration to the striatum is reduced in patients with HD (97). However, evidence of altered NPC production and neuroblast migration in the human brain following the onset of injury or neurodegenerative disease remains controversial. For example, there is divergent evidence regarding altered NPC production in the SVZ of patients with Parkinson's Disease (PD), with some research claiming decreased NPC production in PD (128) and others failing to find any difference between PD patients and healthy controls (129). Similar to the research describing human adult neurogenesis that was discussed in section Controversy Over Human SVZ and RMS, these studies have small numbers of heterogeneous patients and were conducted in limited postmortem tissue samples. The current available research makes it difficult to generalize these findings to the human population and form definitive conclusions regarding the extent of altered neurogenesis in the human brain following the onset of injury and neurodegenerative disease.

These studies conducted across species highlight the potential of the SVZ to act as an endogenous source of replacement for lost or damaged neurons in response to diverse acquired brain injury and neurodegenerative disease arising in the adult brain. Observations of attempted endogenous neuroregeneration increased interest in the potential to harness endogenous stem cells as a tool to promote brain repair (36, 130). These examples of inherent repair mechanisms within the brain have been the inspiration for the biomaterial and tissue engineering strategies that attempt to manipulate or augment this endogenous neurogenesis that will be discussed in this review.

## Current Strategies to Augment and Redirect Endogenous Neurogenesis

Here we will review the experimental approaches that currently exist to augment and redirect SVZ neuroblast migration, including various pharmacological approaches and acellular biomaterial scaffolds. We also discuss a tissue-engineered RMS, a newly developed technology that is predicted to facilitate sustained redirection of endogenous neuroblasts from the RMS and into neuron-deficient regions.

### Pharmacological Approaches: Neurotrophic Factors and Signaling Peptides

**Figure 2:**
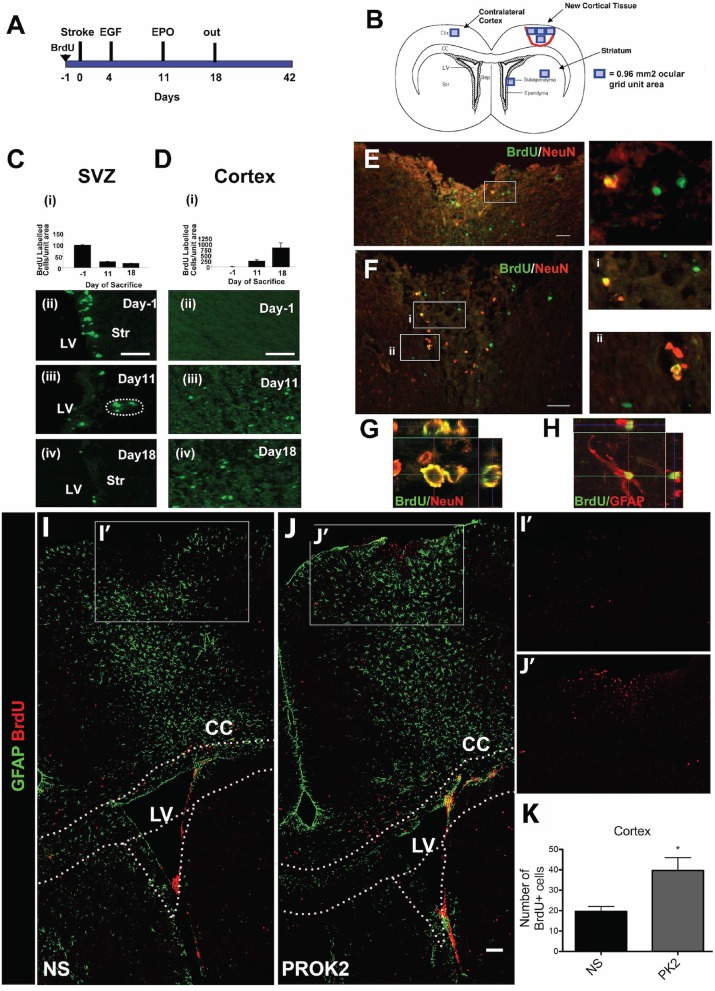
Pharmacological and signaling peptide approaches to augment endogenous NPC proliferation and migration. **(A–H)** Intraventricular infusion of EGF followed by EPO and **(I–K)** injection of recombinant Prokinectin 2 (PROK2) into the injured cortex. **(A)** Experimental design utilized by Kolb et al. (137). Rats received BrdU injections 1 day prior to stroke, followed by EGF on days 3 and 4 following stroke and EPO on day 11 following stroke. **(B)** Schematic illustration depicting the four regions (indicated by squares) in which BrdU-positive cells were counted. **(C,D)** Quantification and corresponding fluorescent images depicting the number of BrdU-positive cells in the SVZ (over the 0.96 mm^2^ area indicated in **B**) and cortex (over the 3.84 mm^2^ area indicated in B) 1 day before stroke (−1) and 11 and 18 days post-stroke following growth factor administration. BrdU-positive cells decreased in the SVZ and increased in the cortex over time. **(E,F)** Fluorescence images depicting BrdU and NeuN double-positive cells in the injured cortex. Insets depict higher magnification images of NeuN/BrdU cells. Confocal images of **(G)** BrdU/NeuN double-positive neurons and **(H)** BrdU/GFAP double-positive astrocytes in the injured cortical tissue. In the experimental design utilized by Mundim et al. (145), mice were administered BrdU for 2 days prior to the injection of either recombinant PROK2 or saline into the cortices of uninjured mice. **(I,J)** Confocal microscopy images and **(K)** quantification reveal a greater quantity of BrdU-positive cells (red) in the cortex following PROK2 administration compared to saline (NS). Scale bars **(C,D)** 50 microns; **(E–G)** 30 microns; **(K)** 100 microns. Ctx, cortex; cc, corpus callosum; LV, lateral ventricle; Str, striatum; NS, normal saline. Reprinted with permission from Kolb et al. (137) for **(A–H)** and Mundim et al. (145) for **(I–K)**. *p < 0.05.

Neurotrophic factors (NTFs) are proteins important for regulating the growth, survival, and plasticity of neurons (131). Increased SVZ neurogenesis has been observed following administration of NTFs to the uninjured and injured rodent brain (Table 1). For example, intraventricular administration of epidermal growth factor (EGF) (134, 135), fibroblast growth factor 2 (FGF-2) (135), erythropoietin (EPO) (140), or nerve growth factor (NGF) and EGF (139) increased SVZ NPC proliferation and/or migration away from the SVZ neurogenic niche in the SVZ of the uninjured rodent brain. This augmented proliferation of SVZ NPCs was also observed after administration of NGF in the form of eye drops (144). The introduction of various NTFs can also further amplify the increased SVZ neurogenesis that occurs following acquired brain injury such as experimental stroke. Augmented proliferation of SVZ NPCs and migration of these cells toward injured brain regions has been observed following intraventricular infusion of EGF (136), EGF and EPO (137) (Figures 2A–H), EGF and FGF-2 (138), VEGF (147), or BDNF (133) into the post-stroke rodent brain. These effects have also been observed after intranasal administration of TGF-1 (146) or subcutaneous (SC) administration of granulocyte-colony stimulating factor (G-CSF) (141), stromal derived factor 1 (SDF-1) (132), or angiopoietin 1 (Ang1) (132) into the post-stroke rodent brain. These studies collectively provide evidence that administration of various NTFs can augment NPC production and neuroblast migration in rodent models of experimental stroke. However, the majority of these studies fail to examine the long-term survival and integration of neuroblasts into regions of injury. While the majority of these studies did provide evidence of short-term functional recovery as a result of NTF administration (132, 133, 137, 141, 146, 147), research demonstrating long-term functional outcomes resulting from these treatment strategies is lacking. It also remains unknown if NTFs will need to be repeatedly administered in order to continually promote functional recovery over time. While the ability of NTFs to augment NPC production and neuroblast migration are essential first steps to promoting endogenous replacement of lost neuronal populations, the current studies utilizing these treatment strategies do not provide compelling evidence that NTF administration alone is an effective method to promote long-term neuronal recovery following stroke in rodents. Additionally, it is important to note that different ligands of the EGFR receptor have different effects on the fate and migratory capacity of SVZ NPCs following brain injury. For example, the ADAM17/tumor necrosis factor alpha (TNF-alpha)/EGF receptor (EGFR) pathway is upregulated following brain injury and has been shown to exert an anti-neurogenic/pro-gliogenic effect on SVZ NPCs (148, 149) and to inhibit neuroblast migration into regions of injury (150). This is in contrast to the evidence presented above demonstrating a neurogenic effect on SVZ NPCs and increased migration into injured brain regions resulting from EGF administration following brain injury (136, 137). This evidence highlights the possibility that certain receptors may have diverse and complicated effects on neurogenesis in the injured brain, such as occurs with the EGFR.

**Table 1:** Pharmacological approaches to augment proliferation and/or migration of SVZ-derived NPCs in the uninjured and injured rodent brain.

**Neurotrophic factor or signaling peptide (alphabetical)**	**Administration**	**Species**	**Type of injury**	***In vivo* treatment effect**	**Evaluation method**	**References**
Ang1	SC administration for 7 days beginning 1 day post-injury	Mice	Experimental stroke (MCAO)	Increased DCX-positive neuroblasts in the injured cortex	DCX	(132)
BDNF	IV administration for 5 days beginning 1 h post-injury	Rats	Experimental stroke (photothrombotic)	Increased DCX-positive neuroblast migration from the SVZ to the ipsilateral striatum but not to the ischemic cortex	DCX	(133)
EGF	IV administration for 6 days	Mice	Uninjured	Increased SVZ NPC proliferation; increased neuroblast migration away from LV walls; differentiation into BrdU/NeuN-positive neurons and BrdU/S100-positive glial cells	lacZ reporter gene, [^3^H] thymidine, BrdU, NeuN, S100	(134)
EGF	IV administration for 14 days	Rats	Uninjured	Increased SVZ NPC proliferation; increased BrdU-positive cells in the striatum 4 weeks post-infusion	BrdU	(135)
EGF	IV administration for 7 days beginning 2 days post-injury	Mice	Experimental stroke (MCAO)	Increased DCX/BrdU-positive SVZ NPC proliferation; migration of DCX/BrdU-positive cells to the injured striatum and maturation into PV-containing interneurons	DCX, BrdU, PV	(136)
EGF and EPO	IV administration of EGF for 7 days followed by EPO for 7 days beginning 4 days post-injury	Rats	Experimental stroke (focal PVD)	Increased BrdU-positive NPC migration from the SVZ to the injured cortex; differentiation into BrdU/NeuN-positive neurons and BrdU/GFAP-positive glial cells in the injured cortex	BrdU, NeuN, GFAP	(137)
EGF and FGF-2	IV co-administration for 3 days beginning 1 day post-injury	Rats	Experimental stroke (MCAO)	Increased BrdU-positive SVZ NPC proliferation	BrdU	(138)
EGF and NGF	IV co-administration for 4 days followed by single infusion of NGF 4 days later	Mice (Aged)	Uninjured	Increased Ki-67-positive SVZ NPC proliferation	Ki-67	(139)
EPO	IV administration for 6 days	Mice	Uninjured	Increased migration of BrdU-positive NPCs from the SVZ to the OB; increased BrdU/TH-positive neurons in the OB	BrdU, TH	(140)
FGF-2	IV administration for 14 days	Rats	Uninjured	Increased SVZ NPC proliferation; increased BrdU-positive cells in the OB 4 weeks post-infusion	BrdU	(135)
G-CSF	SC administration for 15 days beginning 1-h post-injury	Rats	Experimental stroke (MCAO)	Increased BrdU-positive SVZ NPC proliferation; subset of cells was BrdU/NeuN-positive	BrdU, NeuN	(141)
IL-15	Single IV administration	Mice	Uninjured	Increased BrdU-positive and DCX-positive SVZ NPCs	BrdU, DCX	(142)
NAME (NOS inhibitor)	Single IV administration	Rats	Uninjured	Increased BrdU-positive cells in the SVZ, RMS, and OB	BrdU	(143)
NGF	Single administration in the form of eye drops	Rats	Uninjured	Increased Ki-67-positive SVZ NPC proliferation	Ki-67	(144)
PKRA7 (PROK2 antagonist)	IP administration for 4 days beginning 1 h post-injury	Mice	Blunt force TBI	Decreased BrdU-positive neuroblast migration from the SVZ to the injured cortex	BrdU	(145)
PROK2	Single intracortical administration	Mice	Uninjured	Increased BrdU-positive cells in the cortex	BrdU	(145)
SDF-1	SC administration for 7 days beginning 1 day post-injury	Mice	Experimental stroke (MCAO)	Increased DCX-positive neuroblasts in the injured cortex	DCX	(132)
TGF-1	IN administration 2 and 24 h post-injury	Mice	Experimental stroke (MCAO)	Increased BrdU-positive cells in the SVZ and injured striatum; increased BrdU/DCX/NeuN-positive cells in the striatum	BrdU, DCX, NeuN	(146)
VEGF	IV administration for 3 days beginning 1 day post-injury	Rats	Experimental stroke (MCAO)	Increased BrdU-positive SVZ NPC proliferation	BrdU	(147)

*SVZ, subventricular zone; NPC, neural precursor cell; OB, olfactory bulb; IV, intraventricular; IP, intraperitoneal; IN, intranasal; SC, subcutaneous; LV, lateral ventricle; EGF, epidermal growth factor; FGF, fibroblast growth factor; EPO, erythropoietin; NGF, nerve growth factor; BDNF, brain-derived neurotrophic factor; TGF, transforming growth factor; G-CSF, granulocyte-colony stimulating factor; VEGF, vascular endothelial growth factor; SDF, stromal derived factor; ang, angiopoietin; PROK2, prokineticin 2; IL, interleukin; NAME, N^ω^-nitro-methyl-L[D]-arginine-methyl ester; NOS, nitric oxide synthase; PVD, pial vessel disruption; MCAO, middle cerebral artery occlusion; PV, parvalbumin; TH, tyrosine hydroxylase; DCX, doublecortin*.

Administration of signaling peptides is another pharmacological approach used to augment NPC production within the SVZ. A wide variety of cytokines have been used to modulate NPC proliferation and differentiation *in vitro*, including interleukin (IL)-1, IL-6, IL-10, IL-15, Leukemia inhibitory factor, and TNF-alpha (151). Importantly, cytokines modulate NPC proliferation *in vivo*. For example, intracortical administration of prokineticin 2 (PROK2) into the cortex of uninjured mice caused an increase in BrdU-positive cells in the cortex (Figures 2I–K), while IP administration of PKRA7 (a PROK2 antagonist) caused a decrease in migration of neuroblasts from the SVZ to the injured cortex following blunt force TBI (145). Additionally, intraventricular administration of IL-15 (142) or an NOS inhibitor (143) increased proliferation of SVZ NPCs. Similar to the administration of NTFs, these results of these experiments demonstrate that signaling peptides can augment NPC production within the SVZ of the injured rodent brain. However, the majority of these experiments have not investigated whether these signaling peptides are efficacious at augmenting NPC production within the SVZ following brain injury, leading to a current lack of evidence indicating whether these cytokines have the ability to promote functional recovery post-injury. Further research is needed before conclusions can be drawn regarding the ability of signaling peptide administration to induce neuronal replacement or promote functional recovery following brain injury.

Importantly, NTFs have been utilized in human clinical trials as a treatment strategy to offset neuronal degeneration resulting from neurodegenerative disease. NTFs including ciliary neurotrophic factor (CNTF), BDNF, insulin-like growth factor 1 (IGF-1), NGF, glial cell line-derived neurotrophic factor (GDNF), and neurturin (NRTN) have been delivered via subcutaneous, intrathecal, intraventricular, and IP routes of administration to patients with amyotrophic lateral sclerosis (ALS), PD, Alzheimer's Disease, and peripheral neuropathies (152). However, although there have been over 3 dozen clinical trials investigating the efficacy of neurotrophic factor administration as a treatment for human neurodegenerative disease, none have demonstrated efficacy at preventing neuronal degeneration and death, indicating that perhaps NTF administration alone may not be beneficial to treat human neurodegenerative diseases (9, 152). One reason may be that the effect of NTF introduction appears to be transient. NTFs may need to be repeatedly administered to continually offset neuronal degeneration occurring in the human brain, making this technique repetitively invasive. Additionally, the optimal dosing and the best timing window in which to administer NTFs following the onset of injury or degeneration within the human brain is still being investigated (152, 153). Further investigation into dosing, timing and route of administration, and patient population are needed to determine whether NTFs are efficacious treatments to alleviate the effects of neurodegenerative disease in the human brain.

### Acellular Biomaterial Strategies

Research suggests that transplanting a biomaterial scaffold *without* the addition of NPCs into an area of injury may support the surrounding brain tissue and promote axon regeneration and neurite formation (154). Several studies have transplanted biomaterials into injured brain regions with the goal of augmenting neuronal regeneration via repair of local, damaged brain cells within these areas of injury (11) and enhancing the effectiveness of cell transplantation and drug delivery (155). There are several reviews describing the ability of these biomaterials to bridge neuronal lesions, promote cell growth, and stimulate axonal outgrowth post-transplantation into regions of neuronal injury (10–12, 37, 154). Just as with biomaterials designed to deliver NPCs to regions of injury, these acellular scaffolds can be coupled with NTFs to further enhance neuronal support and regeneration upon transplantation (11, 37, 154, 156). These experiments have used biomaterials to repair local neuronal damage, but have not sought to manipulate endogenous neurogenesis to aid in potential repair.

**Figure 3:**
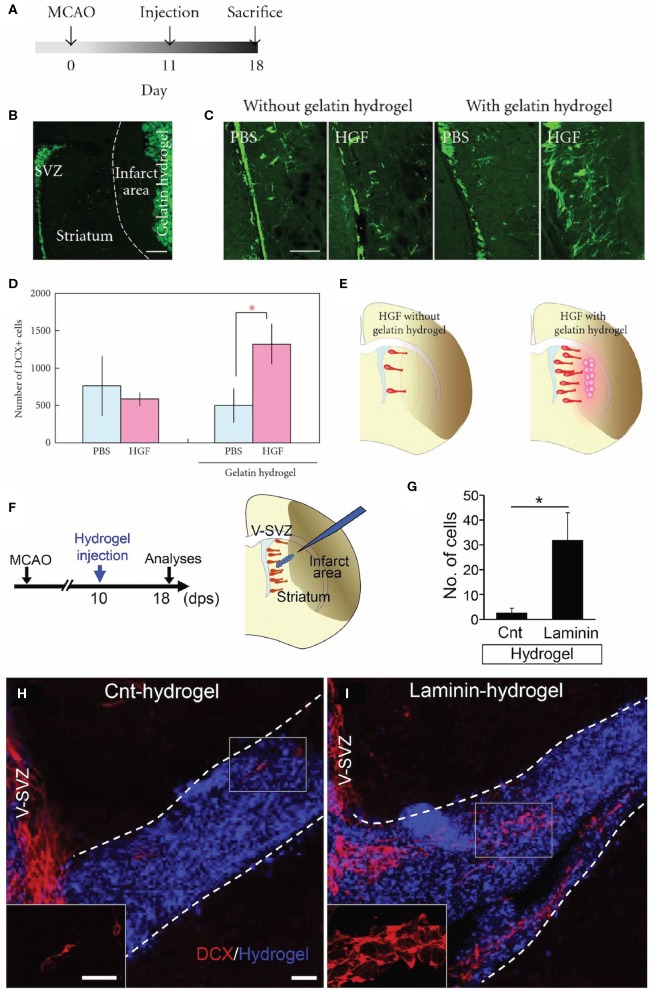
Acellular biomaterial approaches to redirect endogenous NPC migration to regions of injury. Experimental results from *in vivo* implantation of **(A–E)** HGF-containing gelatin hydrogel or **(F–I)** laminin-rich hydrogel. **(A)** Experimental design utilized by Nakaguchi et al. (159). **(B)** DCX-positive (green) cells in the SVZ and gelatin hydrogel injected into the striatum of a coronally-sectioned rat brain. **(C)** Coronal brain sections depicting a higher quantity of DCX-positive cells (green) in the striatum elicited by injection of HGF-containing hydrogel compared to a PBS-containing hydrogel or gelatin hydrogel alone. **(D)** Quantification of DCX-positive cells in the ipsilateral striatum (seen at least 50 microns from the ipsilateral SVZ). **(E)** Schematic illustrating that the HGF-containing hydrogel was more efficacious at recruiting new neurons to the injured striatum compared to injection of HGF alone. **(F)** Experimental design utilized by Fujioka et al. (104). Schematic illustrates the injection of a self-assembling laminin-rich hydrogel into the striatum following experimental stroke. **(G)** Quantification and **(H,I)** confocal microscopy images of DCX-positive cells (red) migrating along hydrogels with and without laminin (blue). A greater quantity of DCX-positive cells is seen migrating along the **(I)** laminin hydrogel compared to **(H)** the hydrogel without laminin (Cnt-hydrogel). Scale bars **(B,C)**: 200 microns; **(E)**: 20 microns. Reprinted with permission from Nakaguchi et al. (159) for **(A–E)** and Fujioka et al. (104) for **(F–I)**. *p < 0.05.

Of particular interest to this review are biomaterial methods that have been developed to enhance *endogenous* neuronal regeneration and/or redirect *endogenous* neuroblasts from the SVZ (Table 2). These approaches are unique in that they attempt to promote neuronal regeneration via manipulation of the brain's intrinsic NPC sources, rather than relying on extrinsic NPCs for repair. For example, biomaterials and nanotechnology have been used as a strategy to enhance the delivery of NTFs into the brain (169, 170). While the use of these technologies *in vivo* has been limited to rodent models, they show promise for enhancing the beneficial effects of NTFs in application to neurodegenerative disorders. In this approach, biomaterials coupled with NTFs are injected into a region of injury to enhance endogenous NPC proliferation and neuroblast migration from the SVZ. Experiments utilizing this approach have demonstrated that coupling NTFs with biomaterials is advantageous over *in vivo* administration of NTFs alone for augmenting endogenous SVZ NPC production and neuroblast migration post-injury. For example, increased proliferation of SVZ NPCs occurred following injection of hydrogels encapsulating IGF-1 (159), hepatocyte growth factor (HGF) (159) (Figures 3A–E), EGF (161), or EPO (162) into the post-stroke rodent brain. The hydrogels encapsulating HGF or EPO also increased migration of neuroblasts into the stroke-injured region (159, 162). Biomaterial approaches are also being developed to promote endogenous neuroblast migration by intercepting the SVZ with implanted hydrogel scaffolds encapsulating NTFs or signaling cues. These constructs are designed to divert migrating neuroblasts from the SVZ into the scaffold and deliver them to alternate locations throughout the brain. For example, implantation of biomaterial hydrogels encapsulating GDNF (160), a BDNF-mimetic (168), graphene coated fibers (166), or laminin 1, aprotinin, NGF, and VEGF (158) spanning from the SVZ into the injured cortex caused NPCs to divert from the SVZ and migrate into the biomaterial implant tract. In other examples, an injectable self-assembling peptide amphiphile carrying the migratory bioactive Tenascin-C derived peptide sequence E_2_Ten-C PA (167) or an injectable self-assembling peptide encapsulating BDNF (157) elicited the diversion of endogenous neuroblasts from the SVZ/RMS and into the injection tract. An N-cadherin-containing gelatin sponge also enhanced neuroblast migration toward regions of injury in the neonatal mouse brain, mimicking the N-cadherin-mediated adhesion interactions between radial glial fibers and neuroblasts during developmental and post-injury migration (165). Another approach builds off of the observation that endogenous neuroblasts can travel along blood vessel scaffolds when migrating to injured brain regions (102, 103, 105, 171–173). This method constructs scaffolds out of laminin, which is a major component of the basement membrane of blood vessels (174). Transplantation of a laminin-rich porous sponge (164), an injectable laminin-rich hydrogel (104) (Figures 3F–I), or a laminin tract with no coupled biomaterials (163) into the injured rodent brain effectively enhanced neuroblast migration into lesioned regions. Collectively, these technologies have demonstrated the rich potential for enhancing and redirecting migration of endogenous neuroblasts. Implantation of a biomaterial scaffold intercepting the SVZ and spanning toward a region of injury may offer greater spatial control over the migration path of neuroblasts emerging from the SVZ than other methods that have been previously designed to augment and redirect neuroblast migration, indicating that this may be the most promising strategy for neuroblast redirection to specific neuron-deficient regions of the brain. Importantly, biomaterial scaffolds that are designed to mimic certain mechanisms with which neuroblasts migrate endogenously throughout the brain (i.e., endogenous signaling mechanisms and endogenous scaffolds) may be particularly effective at eliciting neuroblast migration into regions of injury. Further investigation of survival, maturity, and synaptic integration of redirected neuroblasts, as well as the ability of these scaffolds to promote functional recovery following injury, is needed before the efficacy of these biomaterial methods can be fully determined.

**Table 2:** Acellular biomaterial strategies to augment proliferation and/or migration of SVZ-derived NPCs in the injured rodent brain.

**Biomaterial approach (alphabetical)**	**Administration or implantation strategy**	**Species**	**Type of injury**	***In vivo* treatment effect**	**Evaluation method**	**References**
β-peptide hydrogel (self-assembling) encapsulating BDNF	Implanted to intercept the SVZ and span toward the cortex	Mice (transgenic NestinCre-ER^T2^:R26eYFP to fluorescently label SVZ NPCs)	Implant tract	GFP/DCX-positive cells in implanted hydrogel; GFP/NeuN-positive cells and GFP/Syn1-positive cells in and at the end of the hydrogel	GFP (permanently labeled in SVZ progeny), DCX, NeuN, Syn1	(157)
Fibrinogen hydrogel containing laminin 1, aprotinin, NGF, VEGF	Implanted to intersect the RMS and span toward the striatum	Rats	Implant tract	DCX-positive cells diverted from the RMS, migrated along implant tract, and entered into ventral striatum (seen at 4-5 weeks post-implantation)	DCX	(158)
Gelatin hydrogel containing IGF-1	Injected into the striatum near the SVZ 11 days post-injury	Mice	Experimental stroke (MCAO)	Increased DCX-positive SVZ NPCs compared to injection of IGF alone	DCX	(159)
Gelatin hydrogel containing HGF	Injected into the striatum near the SVZ 11 days post-injury	Mice	Experimental stroke (MCAO)	Increased migration of DCX-positive neuroblasts from the SVZ to the injured striatum compared to injection of HGF alone	DCX	(159)
Gelatin-HPA (with and without CMC-Tyr) encapsulating GDNF	Implanted to intercept the SVZ and span toward the cortex	Rats	Implant tract	DCX-positive cells migrated along implant tract at 7 but not 12 days following implantation	DCX	(160)
HAMC hydrogel containing EGF or PEG-EGF	Placed epi-cortically over stroke region 4 days post-injury	Mice	Experimental stroke (induced by ET-1)	Increased Ki-67/DCX-positive SVZ NPC proliferation	Ki-67, DCX	(161)
HAMC hydrogel containing EPO	Placed epi-cortically over stroke region 4 and 11 days post-injury	Mice	Experimental stroke (induced by ET-1)	Increased Ki-67/DCX-positive SVZ NPC proliferation; increased NeuN-positive neurons in the injured cortex	Ki-67, DCX, NeuN	(162)
Laminin tract	Injected spanning from the RMS to the lesion 5 days post-injury	Rats	Cortical lesion induced by injection of ibotenic acid	Increased DCX-positive cells along the length of the tract as well as in the lesion compared to control injection; lesion contained DCX/GFAP-positive cells and DCX/NeuN-positive cells	DCX, GFAP, NeuN	(163)
Laminin-rich injectable hydrogel	Injected into the injured striatum 10 days post-injury	Mice	Experimental stroke (MCAO)	Increased DCX-positive neuroblasts migrating on laminin-containing hydrogel compared to control hydrogel; neuroblast chain migration on laminin hydrogel only	DCX	(104)
Laminin-rich porous sponge	Implanted into injured cortex 3 days post-injury	Mice	Cryogenic cortical injury	Increased DCX-positive and GFAP-positive cells within lesion site	DCX, GFAP	(164)
N-cadherin-containing gelatin sponge	Implanted into the injured cortex 3 days post-injury	Mice (neonatal)	Cryogenic cortical injury	Increased DCX-positive neuroblasts in injured region compared control; increased SVZ-derived NeuN-positive neurons in the injured cortex 28 days post-injury	DCX, NeuN	(165)
PEM-PCL electrospun scaffold (graphene coated)	Implanted to intercept the SVZ and span both dorsally and ventrally	Rats	Implant tract	DCX-positive neuroblasts diverted from the SVZ and migrated along the scaffold	DCX	(166)
Peptide amphiphile (self-assembling) carrying a Tenascin-C signal (E_2_Ten-C PA)	Injected to intersect the RMS and span toward the neocortex	Mice	Implant tract	DCX-positive cells diverted from the RMS and migrated along implant tract (seen at 7 days post-injection)	DCX	(167)
PCL electrospun scaffold encapsulating BDNF-mimetic	Implanted to intercept the SVZ and span toward the cortex	Rats	Implant tract	DCX-positive neuroblasts diverted from the SVZ and migrated along the implant tract toward the cortex; SMI32-positive neurites observed in the scaffold 21 days post-implantation	DCX, SMI32	(168)

*SVZ, subventricular zone; NPC, neural precursor cell; IGF, insulin-like growth factor; HGF, hepatocyte growth factor; HAMC, hyaluronan and methylcellulose; EGF, epidermal growth factor; PEG-EGF, poly(ethylene glycol)-modified EGF; EPO, erythropoietin; HPA, hydroxyphenylpropionic acid; CMC-Tyr, carboxymethylcellulose-tyramine; GDNF, glial cell-line-derived neurotrophic factor; ET-1, endothelin-1; PCL, poly ε-caprolactone; PEM, polyethyleneimine; BDNF, brain-derived neurotrophic factor; NGF, nerve growth factor; VEGF, vascular endothelial growth factor; Syn1, synapsin 1; DCX, doublecortin; GFAP, glial fibrillary acidic protein; GFP, green fluorescent protein; MCAO, middle cerebral artery occlusion*.

### Living Scaffolds

**Figure 4:**
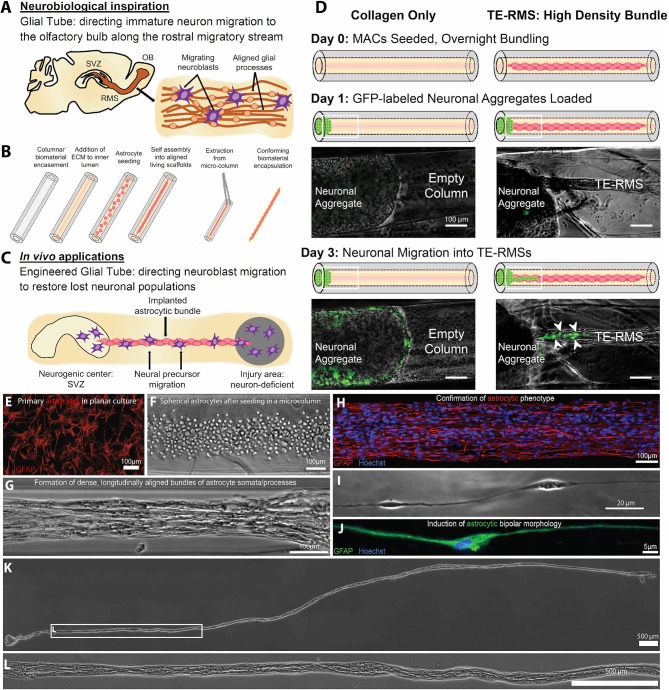
A tissue-engineered rostral migratory stream (TE-RMS) for directed neuronal replacement following brain injury. Schematic illustration of **(A)** the rodent rostral migratory stream comprised of aligned astrocytes, **(B)** the TE-RMS fabrication process, and **(C)** predicted *in vivo* applications of this technology. **(D)** GFP-transduced cortical neuronal aggregates were seeded at one end of hydrogel columns containing either fully formed TE-RMSs or collagen only. Immature neurons migrated out of cortical aggregates and along TE-RMSs, but not along acellular collagen-only columns. White arrows indicate migrating immature neurons. Fluorescence microscopy and phase contrast images showing **(E)** the GFAP-positive astrocytes with stellate morphology used to fabricate TE-RMSs, **(F)** the high density of astrocytes shortly after seeding into a collagen-containing microcolumn during TE-RMS fabrication, **(G)** self-assembly of astrocytes into dense longitudinal bundles during TE-RMS formation, **(H)** maintenance of astrocyte alignment and bipolar morphology post-extraction from the microcolumn, and **(I,J)** individual aligned astrocyte processes within microcolumns. **(K,L)** Phase contrast images demonstrating that centimeter-scale TE-RMSs (>1.5 cm) maintain integrity when extracted from the hydrogel microcolumn. Scale bars **(D–H)** 100 microns; I: 20 microns; **(K,L)**: 500 microns. Adapted with permission from O'Donnell et al. (40) for **(A–D)** and Winter et al. (39) for **(E–L)**.

“Living scaffolds” are a tissue engineering regeneration strategy based on the use of living cells that are encapsulated within a preformed three-dimensional structure and can be designed to facilitate axonal pathfinding, replace circuitry, or direct neuronal cell migration (40, 175, 176). Our laboratory has developed the first implantable living scaffold that emulates the glial tube of the RMS (Figure 4). These tissue-engineered rostral migratory streams (TE-RMSs) are fabricated by promoting the self-assembly of astrocytes seeded into hydrogel microcolumns that spontaneously re-configure into networks of bipolar, longitudinally-aligned bundles (38, 39) (Figures 4A–B). These bundles provide the scaffolding network through which immature neurons can migrate (Figure 4C). TE-RMSs are fabricated in hydrogel microcolumns with a diameter of <350 microns in which cells self-align into cable-like structures of process-bearing astrocytes enriched in GFAP and measuring 50–150 microns in diameter (38, 39) (Figures 4E–J). We have created TE-RMS scaffolds up to 3 cm long, with no theoretical limit to the length (Figures 4K–L). This tissue engineering approach leverages the brain's natural mechanism for sustained neuronal replacement by replicating the structure and function of the RMS, setting this technology apart from biomaterial strategies that have been created to enhance endogenous neuronal replacement throughout the brain.

The TE-RMS has the potential to act as a living “implantable highway” to reroute immature neurons from the SVZ to diverse brain regions, offering an approach to manipulate neuronal migration throughout the brain via the brain's own mechanism for neuronal replacement throughout adulthood. We predict that this technology will be particularly advantageous above other biomaterial methods due to the hypothesized ability of neuroblasts to actively communicate with the living astrocytes comprising our scaffold, replicating the Slit/Robo signaling and numerous other cellular interactions that facilitate neuroblast migration along the endogenous RMS (8, 177, 178). Unlike most cell transplant techniques, TE-RMSs are designed to be stable, long-term implants that will provide sustained delivery of neuroblasts to neuron-deficient regions over time. That is, the stable structure of the TE-RMS is predicted to reliably redirect the migration of endogenous NPCs from the SVZ to injured and/or degenerated brain regions over time. The characteristic diameter of these scaffolds is suitable for diffusion-based mass transport from the host vasculature, providing benefits for acute and chronic survival of TE-RMS implants within the brain. We hypothesize that this advanced technology will act as an exogenous migratory stream, enabling endogenous neuroblasts to navigate from one region to another, mature into appropriate end-target neuronal phenotypes, and synaptically integrate with host circuitry.

The overall success of the TE-RMS as an implantable technology relies on its ability to replicate *all* known functional aspects of the RMS—that is, to direct neuronal migration, support maturation, and potentially prime immature neurons for integration into circuitry via bidirectional communication with the astrocytes comprising the living scaffold. Studies to date have shown *in vitro* chain migration of immature neurons through the TE-RMS, but not through biomaterial (collagen) control columns (40) (Figure 4D). Future studies in our laboratory will quantify the expression of astrocytic surface markers of cells comprising the TE-RMS and compare that with surface expression of astrocytes within the endogenous RMS. We will then evaluate the ability of this implantable microtissue to direct and sustain immature neuronal migration, influence cell fate determination, and promote neuronal regeneration throughout diverse brain regions in adult rodents. Future implantation studies will evaluate subjects for at least 6 months to assess the chronic fate of the new neurons as well as the fate of the glial cells comprising the TE-RMS. Importantly, future studies will explore the ability to fabricate these constructs with astrocytes derived from adult human mesenchymal stem cells [for examples, see (179)]. The ability to fabricate the TE-RMS from an adult human stem cell source will lay the foundation for a new approach to create patient-specific autologous implants, minimizing the risk for rejection after transplantation.

## Challenges to Overcome and Potential Future Applications

**Figure 5:**
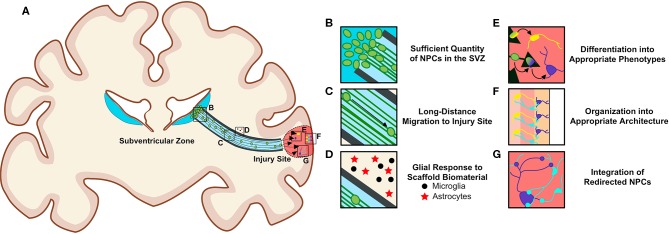
Requirements for the successful redirection of endogenous neuronal stem cells with biomaterial and tissue-engineered scaffolds. **(A)** Schematic depicting a nondescript tissue-engineered scaffold implanted into a gyrencephalic brain spanning from the SVZ to a region of injury. In order for scaffolds to successfully re-direct endogenous NPCs, there **(B)** must be a sufficient quantity of NPCs in the SVZ, **(C)** neuroblasts must migrate long distances within the scaffold, and **(D)** biomaterials composing the scaffolds should not elicit a detrimental immunogenic foreign body response from local and/or recruited glial cells surrounding the implant. Redirected NPCs must **(E)** differentiate into appropriate phenotypes, **(F)** arrange into appropriate architecture, and **(G)** integrate into existing circuitry once they reach their new destination within the brain. Scaffolds may need to be adapted to promote maturation into multiple neuronal phenotypes if differentiation cues at destination are insufficient. Consideration must also be given to how a diseased environment may affect the health of the redirected NPCs. Long-term biocompatibility of the scaffolds with surrounding brain tissue, appropriate degradation rate, and optimal time window for implantation must also be considered. Original figure created by Dayo Adewole, Department of Bioengineering, University of Pennsylvania.

There are notable challenges to overcome to successfully utilize biomaterial and tissue-engineered technologies to harness endogenous NPCs for repair purposes (Figures 5A–G). To be effective treatments to offset neuronal loss, these technologies must deliver a sufficient quantity of NPCs to neuron-deficient brain regions (Figures 5B,C). Redirected NPCs must also exhibit proper neuronal differentiation, maturation, and integration with local neuronal circuitry (Figures 5E–G). Additionally, consideration must be given to how a diseased neuronal environment may affect the biomaterials that compose these technologies, and how the long-term presence of these scaffolds within the brain may alter the environment and affect neuronal and/or glial function (Figure 5D). The biocompatibility of all foreign materials must be confirmed, the timing of implantation and degradation must be determined, and rigorous testing, imaging, and analysis will be necessary as research progresses toward clinical utilization of these technologies.

The majority of the tissue-engineered scaffolds discussed in this review rely on the presence of NPCs within the SVZ to function (Figure 5B). For these technologies to translate into potential treatments for neurodevelopmental disorders, neurodegenerative diseases, and various brain injuries, they must reliably deliver large numbers of endogenous NPCs over long distances into neuron-deficient regions at a rate sufficient to combat neuronal loss (Figure 5C). This inherently requires that the SVZ contains a large enough NPC precursor population to supply sufficient cell numbers at the time of scaffold implantation, regardless of the age of the patient. Whether neuronal proliferation in the human SVZ becomes altered in the injured or degenerated brain remains unknown. As discussed previously, while some research has provided evidence that SVZ NPC proliferation and neuroblast migration are increased in the injured and degenerated human brain (124, 125, 127), these findings remain controversial. Increased SVZ proliferation would increase the pool of NPCs that could be harnessed for redirection, providing tissue-engineered scaffolds the necessary cell source for diversion from the SVZ into neuron-deficient regions. However, if injury or disease impairs proliferation within the SVZ, then the capacity of these scaffolds to deliver NPCs to atrophied regions would be diminished. If the SVZ lacks NPCs, then scaffolds must be modified to induce NPC proliferation within this niche such that it contains sufficient cell numbers or be combined with a complimentary strategy to increase SVZ capacity. Current knowledge regarding how neurogenesis is regulated in the rodent SVZ (78, 80) will instruct future research investigating how endogenous neurogenesis may be regulated in the human SVZ. Mechanisms instructing human SVZ neurogenesis will be critical to uncover in order to understand how neurogenesis in the human SVZ could be upregulated simultaneously with the implantation of these technologies. If the number of proliferative cycles for SVZ NPCs turns out to be limited, causing injury-induced increases in NPC production to be offset by long-term reductions in neurogenesis later in life, then strategies to create or induce an infinite *in vivo* stem cell niche should be explored. Additionally, if future research reveals a complete lack of neurogenesis in the adult human brain, then the tissue-engineered scaffolds discussed herein can be modified to include their own pool of NPCs. That is, a tissue-engineered “neurogenic niche” can be included to provide an NPC source for sustained delivery into a lesion by the TE-RMS. Such a transplantation strategy will be designed to slowly deliver the encompassed NPCs into neuron-deficient regions, making this approach advantageous compared to traditional exogenous cell transplantation techniques that overwhelm neuron-deficient regions with large numbers of new neurons simultaneously.

Additionally, the success of these technologies relies on the ability of neuroblasts directed from the SVZ to regions of injury or degeneration to differentiate into subtype- and region-specific appropriate phenotypes (Figure 5E). There is preclinical evidence that enhancing SVZ neuroblast migration into a striatal lesion following MCAO in mice leads to differentiation of neuroblasts into destination-appropriate striatal projection neurons, integration into existing circuitry, and improved functional recovery (110). However, despite this evidence, the capacity for SVZ neuroblasts to assume diverse phenotypes appropriate for replacement and repair throughout the brain, and whether these processes will become altered in the diseased or injured human brain, remains unknown. A more comprehensive understanding of the cellular and molecular controls over neuronal differentiation both during development and in the adult brain is required to understand if this will be possible (180). Scaffolds may need to be modified to encompass additional signals and cues that will promote successful neuronal migration, maturation, differentiation, and integration into various brain regions. Modification may be required for each specific phenotype of neuron that needs to be generated. Here, our TE-RMS scaffolds have the potential to be further engineered to provide astrocyte-based cues to direct the time course of neuronal differentiation and subtype specification. We envision that these scaffolds may need to be designed to contain specialized factors and/or cues depending on which area of the brain requires NPCs and the neuronal subtype requiring replacement. If these technologies are modified to include additional soluble factors (such as NTFs suspended in the ECM encapsulation), it will be critical to determine the dose, total inclusion volume, and release rate over time. In addition, appropriate methods that allow for accurate *in vivo* phenotype identification of neurons need to be developed and validated to ensure that newly migrated cells develop into mature, region-appropriate neurons. Thus, in addition to investigating whether these technologies are capable of modulating proliferation and migration of cells within the SVZ, it is important that future research will be directed at whether these technologies have the ability to direct cell fate, as this will be a true determinant of whether these technologies can successfully promote functional neuronal replacement.

In addition, appropriate integration and connectivity of neurons will be essential for proper function within new brain regions (Figures 5F,G). Strategies to promote appropriate axonal extension and proper axon pathfinding of introduced neuroblasts may need to be built into these technologies or introduced in parallel with implantation of tissue-engineered scaffolds. In this respect, scaffolds that promote gradual introduction of neuroblasts, mimicking development-like processes, may be more efficacious than introduction of large numbers of neuroblasts all at one time. Indeed, if neuroblasts are introduced gradually over time, then conditions may be more favorable for proper assimilation into local networks. However, this will need to be rigorously evaluated and verified. Additionally, gradual introduction of NPCs into specific brain regions may not be efficacious if long-distance connections need to be simultaneously re-established within the brain. In this case, other complementary technologies may be used in parallel to repair lost connectivity. For example, our laboratory has previously developed micro-tissue engineered neural networks (micro-TENNs), which are implantable living scaffolds composed of neurons and preformed long-projecting axonal tracts encased within hydrogel microcolumns designed to recapitulate the structure and function of specific neuronal-axonal pathways (176, 181–186). Parallel implantation of micro-TENNs, which are designed to reconstruct damaged or lost long-distance axonal circuitry via synaptic integration within host tissue, and TE-RMSs may synergistically act to replace local neurons and re-establish long-distance axonal connections across damaged brain regions. Moreover, implantation of scaffolds may be more effective when combined with other therapies to provide injury-specific rehabilitation of individual brain regions.

In addition to the establishment of proper integration, another challenge will be determining the effect that the environment may have on the neuroblasts that migrate to regions of injury or degeneration. Even if implanted scaffolds successfully facilitate neuroblast relocation to degenerated and/or diseased brain areas, it is still unknown how these aberrant environments may affect the health and integrity of these cells (36). There will likely be many signals from the local neuronal environment that could impair beneficial differentiation and integration of redirected neuroblasts. For example, previous research has demonstrated that transplanted fetal mesencephalic dopaminergic neurons into the brains of human patients with Parkinson's Disease developed alpha-synuclein-positive Lewy bodies (187). Therefore, it could be assumed that *endogenous* neuroblasts redirected to the substantia nigra in patients with PD may also develop Lewy bodies. It is highly plausible that neuroblasts redirected to areas of injury and degeneration could take on aberrant phenotypes caused by signals that are present in these diseased environments. The addition of neuroblasts to certain regions of the brain could also cause additional abnormal functioning of the brain microenvironment, leading to seizures, pain, or new neurological deficits.

Consideration must also be given to whether the presence of foreign biomaterials in the brain will cause changes in inflammation or aberrant functioning of resident glial cells (Figure 5D). Research has demonstrated that the process of inflammation and the phenotype of microglia and macrophages are critical in determining the extent of regeneration that can occur post-injury in the CNS (188). It is important to consider how microglia and macrophages influence inflammation in the brain after injury, and how tissue-engineered technologies may need to be modified such that they secrete factors to facilitate glial cell phenotypes that will not be detrimental to the integration of newly migrated neuroblasts into local circuitry (188).

Timing of implantation will be another important determinant of whether these technologies will effectively replace lost neuronal populations. Introduction of scaffolds in the acute phase following injury and/or degeneration may limit further degeneration by promoting regenerative effects prior to major cell loss or damage. However, introduction of scaffolds immediately or soon after injury may expose these implants to a hostile microenvironment induced by the effects of resident inflammation and necrosis, potentially altering the ability of scaffolds to integrate, the time course of scaffold degradation, and the ability of scaffolds to redirect neuroblasts and promote cell survival. Therefore, if the goal is neuronal replacement, then scaffold implantation may be the most successful when introduced during a sub-acute or intermediate phase following brain injury and/or degeneration after factors contributing to acute toxic and detrimental environments have had a chance to subside (175). Delaying scaffold implantation until the secondary injury environment has stabilized may maximize the regenerative and restorative capability of these technologies. Determination of the optimal time window for scaffold implantation following each specific injury or disorder will be critical to appropriately select patients most likely to benefit from these therapies.

Importantly, it is also unknown whether biomaterial and tissue-engineered scaffolds discussed herein have long-term compatibility with human brain tissue. Consideration must be given to how specific biomaterials may react to the brain microenvironment *and* how the brain microenvironment may react to the presence of foreign biomaterials. These technologies must be rigorously tested before implantation into the human brain can become conceivable. Longitudinal implantation studies must be conducted in multiple animal models, likely including gyrencephalic species to at least approach the neuroanatomy and scale of the human brain. These studies should be repeated by multiple research groups to ensure replicability. Scaffold removal would likely cause undesirable tissue damage, so it may be beneficial to design scaffolds to ultimately biodegrade rather than requiring removal. Consideration must therefore be given to when and how scaffolds will biodegrade, and whether the byproducts will be safe for the brain to metabolize and excrete. The biomaterial components of our living scaffolds, including the TE-RMS, are designed to chaperone delivery and then be gradually reabsorbed, leaving only the anatomically-inspired living component (38, 39, 176, 181–186). TE-RMS scaffolds may require optimization of ECM-based encapsulation techniques to maximize stability and integration while minimizing the microtissue footprint.

There are additional challenges associated with the clinical implementation of regenerative medicine products, particularly biomaterial-based devices and tissue-engineered medical products. Standardized protocols need to be developed for scaffold implantation that take into consideration the timing, location, and logistics of implantation, and can be adapted to diverse patients with heterogeneous injuries and/or degeneration. Additionally, it will be critical to optimize scaffold dimensions, delivery devices, and injection rate to ensure preservation of brain tissue surrounding the implant. Each component composing various scaffolds will need to undergo certification of biocompatibility and lack of any cytotoxic sub-components. Studies will be conducted to examine possible toxicity and immune reactions resulting from the long-term presence of these materials in the human brain. All biomanufacturing procedures must adhere to the FDA current good manufacturing practice (cGMP) regulations. Rigorous regulatory requirements will include quality assurance protocols, including health and potency following storage and distribution of biomanufactured products, particularly those that include live cells. There will be stringent preclinical and clinical testing requirements to ensure product safety and efficacy that will require significant time and expense to achieve.

Finally, the development of reliable, non-invasive imaging methods would be useful to allow for visualization of scaffolds within the brain post-implantation. Once in the brain, it will be critical to visualize them at regular intervals to ensure they are degrading appropriately (if relevant) and not causing aberrant effects. Ideally, imaging methods should have the capacity to elucidate whether the scaffolds are working to appropriately redirect endogenous neuroblasts to neuron-deficient brain regions. As these challenges indicate, there are many milestones to overcome before tissue-engineered technologies will be safe for delivery into the human brain.

As the development of biomaterial and tissue engineering strategies to redirect endogenous neuroblasts advances, uncertainty regarding the existence of the adult human SVZ and RMS raises questions regarding whether these technologies *realistically* have the potential to redirect endogenous neuroblast migration in the human brain. If future research reveals that there is a very low quantity of endogenous NPCs that exist within the adult human SVZ, then biomaterial scaffolds designed to redirect endogenous NPCs are unlikely to promote neuronal repair on their own. Complementary strategies such as gene therapy or stem cell implants to rejuvenate the SVZ may be explored in parallel (189–193). Alternatively, future research may reveal that the human SVZ contains a sufficient quantity of NPCs to be redirected but that the RMS is non-existent or ineffective at eliciting neuronal migration within the adult brain. If lack of a functional RMS is the only factor preventing neuroblast migration, then perhaps replacement of an RMS-like structure is all that would be needed in order to elicit neuroblast migration out of the SVZ in the adult brain. We recognize that implantation of these technologies will most likely need to be coupled with a significant increase in endogenous neurogenesis in the human SVZ in order for endogenous neuroblasts to be successfully redirected. However, lack of knowledge regarding the existence and function of the SVZ and RMS within the adult human brain should not prevent the field from pursuing the development of technological repair strategies that build off of the existence of endogenous NPCs in the adult human brain.

With this in mind, we predict that tissue engineering and biomaterial strategies that redirect endogenous neuroblasts throughout the brain have the potential to serve as beneficial treatments for a variety of neurodevelopmental and neurodegenerative diseases as well as acquired brain injuries. We recognize that discussion of potential clinical utilization of these strategies is currently quite speculative. However, it is important to acknowledge the diverse potential applications envisioned for human patients while these technologies are still in the process of being developed, as the intended clinical applications will certainly influence key design decisions. Examples of diseases and disorders across the human lifespan for which these technologies may eventually be applicable are outlined in Table 3. A wide variety of developmental disorders such as fetal alcohol syndrome disorders (194), high-functioning autism spectrum disorders (195, 196), Tourette syndrome (197), attention-deficit/hyperactivity disorder (198–201), 22q11.2 deletion syndrome (202), childhood absence epilepsy (203), and hypomyelination with atrophy of the basal ganglia and cerebellum (204, 205) are characterized by thinning of specific brain regions. It is possible that implantation of tissue-engineered scaffolds into the developing brains of infants and children leading to controlled redirection of endogenous neuroblasts into relevant neuron-deficient regions has the potential to partially combat gray matter deficiencies that occur in these diverse developmental disorders. If redirected neuroblasts are able to successfully mature and integrate into functional neuronal circuits within neuron-deficient regions, then perhaps this could mitigate some of the developmental defects that are characteristic of these disorders. While introduction of neuroblasts into gray matter-deficient regions would not be a cure for these disorders, it may offset or lessen some of the symptoms that arise specifically due to gray matter loss in specific developing brain regions.

**Table 3:** Human diseases, disorders, and injuries that are characterized by neuronal loss in focal brain regions.

**Injury, disease, or disorder**	**Brain region lacking neurons**	**Relevant references consulted**
**Developmental disorders**		
Fetal alcohol syndrome disorders	Cortical thinning in the bilateral middle frontal lobes, lateral and inferior temporal and occipital lobes, pre- and post-central areas	(194)
High-functioning autism spectrum disorders	Cortical thinning in the left temporal and parietal cortices	(195, 196)
Tourette syndrome	Cortical thinning in frontal and parietal lobes (specifically in ventral sensory and motor regions)	(197)
Attention-deficit/hyperactivity disorder	Cortical thinning in frontal, parietal, and temporal lobes (specifically in pars opercularis, medial temporal cortices, medial and superior prefrontal and precentral regions)	(198–201)
22q11.2 deletion syndrome	Cortical thinning in superior parietal cortices, right parietooccipital cortex, and bilateral pars orbitalis	(202)
Childhood absence epilepsy	Cortical thinning in left orbital frontal gyrus and bilateral temporal lobes	(203)
Hypomyelination with atrophy of the basal ganglia and cerebellum	Small putamen and caudate nucleus; cerebellar atrophy	(204, 205)
**Neurodegenerative diseases**		
Parkinson's disease	Degeneration of substantia nigra pars compacta	(206, 207)
Huntington's disease	Degeneration of striatal medium spiny neurons in the caudate and putamen	(208, 209)
Amyotrophic lateral sclerosis	Atrophy of upper motor neurons in the motor cortex and lower motor neurons in the brainstem and spinal cord	(210, 211)
**Acquired brain injury**		
Focal ischemic stroke	Various localized brain regions	(212–216)
Focal traumatic brain injury	Various localized brain regions	(217–221)

There are also several neurodegenerative disorders which may benefit from the implantation of the tissue-engineered scaffolds discussed herein. Although disorders of the aging brain are complex and not fully understood, it is known that certain neurodegenerative disorders such as PD (206, 207), HD (208, 209), and ALS (210, 211) are partially characterized by atrophy and neuronal loss in specific brain regions. While there is evidence that neuronal degeneration in PD, HD, and ALS is not confined to these specific regions (206, 208, 209, 222), the most *prominent* and *persistent* neuronal degeneration resulting from these disorders is focal. Recent research has turned to tissue engineering strategies as a means to promote functional brain recovery following injury and degeneration (12, 175, 176, 223). Neurodegenerative disorders characterized by region-specific degeneration, including PD, HD, and ALS, may benefit from implantation of tissue-engineered scaffolds designed to redirect neuroblasts from the SVZ and explicitly deliver them into degenerating regions. The TE-RMS scaffolds being developed by our laboratory, which are specifically designed to provide sustained delivery of immature neurons over time, may prove to be a beneficial strategy to offset the continual degeneration that occurs as these diseases progress. If neurons could be continually replaced at the same rate as they are degenerating, then perhaps this could prolong the function of deteriorating brain regions and lessen some of the cognitive and behavioral phenotypes associated with neurodegenerative disorders.

Biomaterial and tissue-engineered scaffolds may also be advantageous treatments for acquired brain injuries that occur throughout the human lifespan, specifically for focal ischemic stroke and focal traumatic brain injury (TBI). Ischemic stroke can occur throughout the brain, leading to localized tissue damage in various regions containing diverse neuronal populations (212, 213). The human populations at the greatest risk for ischemic stroke are the elderly, fetuses, and newborn infants (214, 215). While less common, acute ischemic stroke also occurs in children and young adults (216). Focal TBI may also occur at any age throughout life (217–221). Similar to the application of these technologies in neurodevelopmental and neurodegenerative disorders, tissue-engineered scaffolds could be used to redirect neuroblasts from the SVZ to regions that have been damaged due to focal ischemic stroke or focal TBI, thereby providing a potential treatment option to offset neuronal loss resulting from these injuries. These examples illustrate the wide range of clinical applications across the human lifespan that could arise from the successful redirection of endogenous neuroblasts via biomaterial and tissue-engineered scaffolds.

As discussed above, there remain several questions regarding how these tissue-engineered technologies would function in the aberrantly developing, degenerating, and injured brain. One concern would be that redirection of neuroblasts could lead to thinning of the brain regions from which these cells were diverted. This might be especially concerning in application to developmental disorders given the immature status of the brain. Perhaps the biggest challenge in understanding the effect that these technologies would have in the context of neural injuries and disorders is that we do not fully understand the mechanisms that regulate the injuries and disorders themselves. Our predictions regarding how these technologies will function could turn out to be accurate in the healthy brain but unreliable in certain instances of the diseased brain. It is possible that these engineered scaffolds could induce unique *in vivo* effects for each clinical application. There are significant challenges to overcome before these technologies can be used clinically.

## Conclusion

The presence of neurogenic niches within the adult brain provides endogenous NPCs that can potentially be harnessed to add neurons in cases of developmental disorders or replace neurons that have been lost due to injury or neurodegenerative disease. There have been substantial efforts to harness these cells for neuronal replacement purposes. The pharmacological, biomaterial, and tissue engineering strategies discussed herein that attempt to augment and/or redirect endogenous neuroblast migration from the SVZ have the potential to offset neuronal loss in diverse regions throughout the brain. Current research has provided evidence that these biomaterial and tissue engineering strategies will have the potential to promote neuronal repair following diverse afflictions including acquired brain injury, developmental disorders, and neurodegenerative diseases that arise throughout life. We predict that the most efficacious strategy to augment and redirect endogenous NPC replacement will build upon the brain's intrinsic mechanisms for neuronal replacement and will have the ability to promote sustained neuronal delivery over time. There are still many challenges to overcome—from ensuring biocompatibility of scaffolds to confirming that they can sufficiently promote neuroblast maturation and synaptic integration—before these newly developed technologies will be able to successfully redirect endogenous NPCs to promote neuronal repair.

## Author Contributions

EP conducted literature review and drafted the manuscript. JO'D, HC, and DC provided significant guidance on manuscript content and critically reviewed the manuscript for important intellectual content. All authors contributed to manuscript revision, read, and approved the submitted version.

## Conflict of Interest

DC is a co-founder of two University of Pennsylvania spin-out companies concentrating in applications of neuroregenerative medicine: INNERVACE, Inc and Axonova Medical, LLC. There are three patent applications related to the methods, composition, and use of micro-tissue engineered glial and neuronal networks, including U.S. Patent App. 15/534,934 titled “Methods of promoting nervous system regeneration” (DC), U.S. Patent App. 15/032,677 titled “Neuronal replacement and reestablishment of axonal connections” (DC) and U.S. Patent App. 16/093,036 titled “Implantable living electrodes and methods for the use thereof” (DC and HC). The remaining authors declare that the research was conducted in the absence of any commercial or financial relationships that could be construed as a potential conflict of interest.
